# Persistence on prostaglandin ocular hypotensive therapy: an assessment using medication possession and days covered on therapy

**DOI:** 10.1186/1471-2415-10-5

**Published:** 2010-03-02

**Authors:** Gregory Reardon, Gail F Schwartz, Sameer Kotak

**Affiliations:** 1 Informagenics, LLC, Worthington, Ohio, USA; The Ohio State University, Columbus, Ohio, USA; 2 Glaucoma Consultants, Greater Baltimore Medical Center; Wilmer Eye Institute, Johns Hopkins University, Baltimore, Maryland, USA; 3 Pfizer Inc, New York, New York, USA

## Abstract

**Background:**

Prior research has demonstrated that medication persistence (continued acquisition of therapy over time) is far from optimal among patients with glaucoma. The purpose of the present study was to evaluate persistence with prostaglandin analogs among glaucoma patients in the first therapy year using a modification of a previously published technique.

**Methods:**

This retrospective analysis of medical and pharmacy claims database included treatment-naive patients dispensed bimatoprost, latanoprost, or travoprost between 1/1/04-12/31/04. "Index agent" was defined as the first agent filled; "index date" was defined as the fill date. Follow-up continued for 358 days. Persistence measures for first therapy year were: (1) whether last fill had sufficient days supply to achieve medication possession at year's end, and (2) number of days for which the index agent was available (days covered). Associations between index agent and medication possession (logistic regression) and days covered (linear regression) were evaluated. Models were adjusted for gender, age, and previous ocular hypertension diagnosis.

**Results:**

7873 patients met inclusion criteria (bimatoprost, n = 1464; latanoprost, n = 4994; travoprost, n = 1415). Medication possession was 28% and days covered was 131 when using the unadjusted (pharmacy-reported) days supply estimates and rose to 47-48% and days covered to 228-236 days when days supply was imputed. Compared to latanoprost, odds of achieving medication possession at first year's end were 26-34% lower for bimatoprost and 34-36% lower for travoprost (p ≤ 0.001 for all comparisons). Days covered in the first year were 21-29 days lower for bimatoprost and 33-42 days lower for travoprost (p ≤ 0.001 for all comparisons). Failure to refill the index agent within the initial 90 days was a strong predictor of poor persistence.

**Conclusions:**

Persistence with ocular prostaglandin therapy remains a problem. Latanoprost users had greater odds of achieving medication possession and had more days covered during the first therapy year.

## Background

More than two million persons in the U.S. have open-angle glaucoma [1]. Studies consistently report that approximately half of patients with glaucoma have been diagnosed or treated for the condition [2-4], and the prevalence of ocular hypertension, which may precede the onset of glaucoma, is considerably higher than that of glaucoma [5-8].

Although it is intuitive that patients with chronic conditions, such as glaucoma and hypertension, must take medication continuously to receive the full benefit of treatment, achieving persistence on pharmacotherapy remains an important challenge [9-14]. For glaucoma therapy, continued acquisition of medication over time, or persistence, has been assessed by a number of methods, including the medication possession ratio [15-17] and survival analysis [18-26]. The consensus of these studies is that persistence is far from optimal for patients receiving topical ocular hypotensive agents.

Greater efforts are clearly needed to improve persistence for what is commonly an asymptomatic condition. A recent study by Wilensky and associates [27] introduced a methodological approach for measuring persistence in glaucoma patients that used days supply as reported on the prescription claim to compare persistence across prostaglandin analogues. In that study, the reported days supply was adjusted upward, then imputed with a conversion factor, to obtain a more realistic estimate of days supply from the fill. The imputed value of days supply for each fill was then used to assess persistence at various points in time by calculating whether the patient had sufficient medication on hand given the days supply available from the last fill prior to that day, and to estimate the number of days in the first therapy year for which they had therapy coverage by summing the imputed days supply for all fills in the first year.

The current study replicated the potentially useful technique developed by Wilensky and associates [27] but with some adjustments to correct for a potential selection bias. For instance, exclusion of early therapy failures in related persistence studies has been criticised for excluding patients who discontinued therapy for reasons such as lack of efficacy or side effects [28].

Herein, we updated the time frame of the patient selection period to 2004 and removed this controversial step for excluding "early failures." Using three alternative methods for imputing days supply from the prescription claim, we evaluated two measures of persistence in the first therapy year: (1) *Medication Possession at the End of Year *(medication possession) and (2) *Days Covered in the First Therapy Year *(days covered). (Note that the term "medication possession" is analogous to the outcome of "persistence" used in Wilensky and associates [27]. The term "days covered" corresponds to the outcome of "adherence" in Wilensky and associates [27].) We compare findings between prostaglandin agents and provide summary estimates for all agents combined.

## Methods

Data were derived from the Ingenix LabRx administrative claims database http://www.ingenix.com. This database contains data from medical, hospital and pharmacy claims that have been submitted to a large, single insurance company. Patients included approximately 10 million U.S. members annually across the U.S., with membership in both commercial health maintenance organisation and preferred provider organisation plans as well as in Medicare-risk plans. The Ingenix database was de-identified in accordance with Health Insurance Portability and Accountability Act requirements prior to being made available for analysis in this study; use of these data in health research is exempt from Institutional Review Board review [29]. Patients who had a prescription dispensed for bimatoprost, latanoprost, or travoprost from 1 January 2004 through 31 December 2004 were screened for inclusion. For each screened patient, all available medical and pharmacy claims data were extracted for the time period 1 January 2003 through 31 December 2005.

Patients were excluded if they: (1) were less than 40 years of age on the index date; (2) were not continuously enrolled for 180 days before and 358 days after the index date; (3) had any topical ocular hypotensive agent dispensed 180 days before the index date; (4) had an ocular hypotensive other than the index agent dispensed on the index date; and (5) had no diagnosis for a glaucoma-related condition (*International Classification of Diseases, Ninth Revision *[*ICD-9*] diagnosis = 365*) for 180 days before the index date. Having previous glaucoma surgery was not a criterion for exclusion. The first agent filled for eligible patients in 2004 was the index therapy, and the date of this initial fill was the index date. Prescription claims were followed for each eligible patient for the first 358 days after the index date, the end-of-year time point used by Wilensky and associates [27].

To estimate persistence across all prostaglandins and to compare individual agents, we adapted the approach by Wilensky and associates [27]. First, *medication possession *was defined dichotomously by whether the date of the last fill of the index prostaglandin within the 358-day follow-up period had sufficient days supply to have medication on hand to achieve possession of medication on day 358, the last day of the therapy year. Medication possession was defined as a dichotomous indicator (yes/no) depending on whether a given patient had medication on hand on the last day of the therapy year (day 358). For instance, to a subject who was dispensed a 30-day supply, 20 days prior to year end (on day 338), would be expected to have a "sufficient days supply" to last through the end of the year (on day 358) and would have a value of "yes" for medication possession. Secondly, we estimated the number of days in the first therapy year for which there was available days supply (*days covered*). This was done by summing the imputed days supply for all index agent fills that occurred between the index date through day 358. For patients who had a summed days supply > 358, the value of days covered was set as 358.

In calculating both measures, we employed three separate imputation methods for days supply to address uncertainty regarding the days supply available in a given prescription fill of a study drug. Each claim included an estimate of days supply as recorded by the pharmacist at dispensing. When including days supply in their study, Wilensky and associates [27] adjusted this value upwards to control for a likely underestimation bias by multiplying by a variable factor of 2.1 for bimatoprost and 2.0 for latanoprost and travoprost. In the current analysis, we applied the exact adjustment factors used by Wilensky and associates [27]. This adjustment factor was estimated by dividing the mean number of days (per mL) between all consecutive fills of each agent by the reported days supply (per mL) reported on the pharmacy claim. Thus, for bimatoprost, the mean days between fills (per mL) was 15.8 days, whereas the reported days supply (per mL) was 7.4 days. The ratio of 15.8/7.4 or 2.1 was used to adjust the mean days supply of a 2.5 mL bimatoprost bottle from the value of 18.5 days reported on the claim to 38.9 days for analysis in that study. We did not recalibrate these adjustment factors with our claims data since the numerator of the adjustment factor (days between fills) would presumably lengthen for patients who were less persistent on a given therapy, thus confounding the adjustment factor with a proxy measure of the outcome being assessed.

To further explore the sensitivity of findings to estimates of days supply we applied three alternative days supply estimates: (1) "Unadjusted" as originally reported on the claim by the pharmacist; (2) "2.0-2.1 Variable Factor" used by Wilensky et al [27] as described above; and (3) "2.0 Constant Factor for all study drugs with a 45-day minimum imputed days supply." For the latter, we chose to set 2.0 as a constant factor across agents for simplicity and set the minimum days supply at 45 days across agents since this agreed with the empirical work of Platt et al. [30], who examined days between actual prostaglandin fills, and since Mick et al. [31] and Fiscella et al. [32] reported *in vitro *expected days supplies of 43 to 56 days across all agents and both studies. For all analyses in this study, results are described separately by replicating each of the three estimates of days supply described above.

For all prostaglandins combined and each agent individually, we estimated medication possession and days covered for each patient. Frequency distributions of patients achieving medication possession were reported for each of the agents and evaluated with a chi-square test of significance. Means were calculated for days covered by a prostaglandin agent, and tests of significance used the *F *test.

To adjust for differences in characteristics among patients receiving different agents, separate regression models were created to adjust for key available factors. For the dichotomous measure, *medication possession*, a logistic regression model was developed using the independent variables prostaglandin agent, gender, age category (40 to 54 years, 55 to 64 years, 65 to 74 years, and 75 years and older), and diagnosis for ocular hypertension/borderline glaucoma during the 180 days prior to and including the index date (*ICD-9 *= 365.00 [preglaucoma], 365.01 [open-angle glaucoma with borderline findings], or 365.04 [ocular hypertension]). For the continuous measure, *days covered*, a linear regression model was developed with the same set of independent variables.

To evaluate whether exclusion of early therapy failures would have affected our findings, we replicated the univariate analyses for our eligible patient cohort against the smaller set of patients who would have remained had this "early failures" exclusion rule been applied.

## Results

Of 42 427 patients who had a prescription fill for one of the index agents during 2004, 7873 were eligible and were retained for analysis (Table 1). Table 2 shows differences in patient characteristics among the treatment cohorts. Prescriptions for bimatoprost, latanoprost, and travoprost accounted for 1464, 4994, and 1415 patients, respectively; 57% of patients were female, and this rate was similar among therapies (p = 0.129). The mean age and the distribution of patients by age categories differed by therapy (p ≤ 0.001 for both). Latanoprost users were oldest (mean = 64 years) followed by those prescribed bimatoprost (mean = 63 years) and then travoprost (mean = 62 years). Although all included subjects were required to have a prior medical service coded for glaucoma, we examined those who also had a service coded for "ocular hypertension" (Table 2) in the previous six months as signal of a new or lower severity glaucoma condition. A substantially larger proportion of latanoprost users (44%) had an "ocular hypertension" diagnosis during the 180-day preindex period followed by users of travoprost (39%) and then bimatoprost (36%; p < 0.001).

**Table 1:** Eligible subjects

Application of exclusion criteria to patient population	Patient count*
Initial population of patients who were	
prescribed a prostaglandin ocular hypotensive	
agent between 1/1/2004-12/31/2004	42 427
Patients < 40 years of age on index prescription	
date	1682
Patients not having continuous enrollment for	
180 days before and 358 days after index date	9595
Patients having another topical ocular	
hypotensive dispensed 180 days before index	
date (ie, not new to glaucoma therapy)	24 823
Patients not having monotherapy on index date	
(ie, had more than one ocular hypotensive agent)	7662
Patients having no diagnosis for any glaucoma-	
related condition (*ICD-9 *diagnosis = 365*)	
for 180 days before index date	9791
Eligible patients for analysis	7873

*Many patients met criteria for more than one of the exclusion rules shown.

*ICD-9 = International Classification of Diseases, Ninth Revision*.

**Table 2:** Patient characteristics

	Bimatoprost	Latanoprost	Travoprost	Total	
Number of patients	1464	4994	1415	7873	P value*
		
	% or mean (SD)				
Female	56%	57%	54%	57%	0.129
Age	62.8 (11.8)	63.7 (12.3)	62.0 (11.8)	63.2 (12.1)	< 0.001
Age group 40 to 54	26%	26%	29%	26%	< 0.001
55 to 64	35%	31%	31%	32%	
65 to 74	20%	21%	23%	21%	
75+	20%	23%	17%	21%	
Ocular hypertension^†^	36%	44%	39%	41%	< 0.001

*Significance from chi-square test (%) or F test (mean). Comparison is between bimatoprost, latanoprost, and travoprost.

^†^Patients were identified with ocular hypertension if they had a medical service with any of these *ICD-9 *diagnostic codes in the 180 days before/on the index date: 365.00 (preglaucoma), 365.01 (open-angle glaucoma with borderline findings), or 365.04 (ocular hypertension).

*ICD-9 = International Classification of Diseases, Ninth Revision*; SD = standard deviation.

For all therapies combined, medication possession was only 28% when using the unadjusted days supply estimate (Table 3). Levels rose to 47 and 48% when the days supply estimates were adjusted with imputation factors. Medication possession varied significantly (p ≤ 0.001) by therapy. Medication possession was equivalent or slightly higher for bimatoprost compared to travoprost. However, medication possession ranged from seven to nine percentage points higher for latanoprost than for bimatoprost across days supply imputations.

**Table 3:** Persistence and days covered estimates

Method of imputing days supply	Bimatoprost	Latanoprost	Travoprost	Total	P value*
**No exclusions applied due to early failure or changes in therapy (base model)**					

	**% or mean (SD)**				

**Medication possession**					
Unadjusted days supply	23%	31%	23%	28%	< 0.001
2.0-2.1 Variable Factor^†^	43%	50%	39%	47%	< 0.001
2.0 Constant Factor/45					
Minimum^3^	43%	52%	41%	48%	< 0.001

**Days covered**					
Unadjusted days supply	119 (80)	141 (89)	108 (76)	131 (86)	< 0.001
2.0-2.1 Variable Factor^†^	218 (115)	239 (118)	197 (116)	228 (118)	< 0.001
2.0 Constant Factor/45					
Minimum^‡^	220 (113)	249 (115)	209 (114)	236 (116)	< 0.001

**Exclusions applied due to early failure or changes in therapy (alternative model)**					

	**% or mean (SD)**				

**Medication possession**					
Unadjusted days supply	34%	44%	35%	41%	< 0.001
2.0-2.1 Variable Factor^†^	60%	66%	56%	64%	< 0.001
2.0 Constant Factor/45					
Minimum^‡^	60%	67%	58%	65%	< 0.001

**Days covered**					
Unadjusted days supply	183 (78)	198 (78)	167 (73)	191 (78)	< 0.001
2.0-2.1 Variable Factor^†^	294 (83)	305 (79)	280 (88)	299 (82)	< 0.001
2.0 Constant Factor/45					
Minimum^‡^	294 (82)	310 (75)	287 (83)	304 (78)	< 0.001

*Significance from chi-square test (%) or *F *test (mean). Comparison is between bimatoprost, latanoprost, and travoprost.

^†^Imputed days supply is unadjusted days supply multiplied by 2.0 (latanoprost, travoprost) or 2.1 (bimatoprost).

^‡^Imputed days supply is unadjusted days supply multiplied by 2.0 (all prostaglandins), with a minimum imputed estimate of 45 days.

SD = standard deviation.

Similar findings were seen when days covered was the outcome (Table 3). For all therapies, the number of days covered in the first therapy year was 131 for the unadjusted days supply estimate. This rose to 228 and 236 days covered when the two other methods of imputation were applied. Days covered varied significantly (p ≤ 0.001) by therapy. Bimatoprost patients had higher days of therapy than travoprost users, ranging from 11 to 21 days. However, latanoprost users had the highest number of days covered, ranging from 21 to 29 days higher than that found for bimatoprost users.

"Early failures" (those subjects who did not refill their prescription within 90 days of starting therapy) accounted for 45 to 63% of the total patient cohort, depending on the days supply imputation method applied. When "early failures" were excluded from the analysis, medication possession among all therapies combined rose by a factor of 1.35 to 1.46 times greater than in the base model (Table 3). Exclusion of "early failures" caused days covered to similarly increase in magnitude by a factor of 1.29 to 1.46 compared to the base model. Differences among agents in the alternative model were still significant.

When adjusted for covariates (Table 4), compared to the reference latanoprost, the odds of achieving medication possession at first year's end were 26 to 34% lower for bimatoprost and 34 to 36% lower for travoprost (p < 0.001 across each imputation; Table 4). Bimatoprost had a somewhat higher odds of achieving medication possession than travoprost when days supply was imputed, but this difference was significant (p = 0.048) only for the imputation method "2.0-2.1 Variable Factor." Patients in age cohorts 55 years of age and older consistently had a higher odds of achieving medication at day 358 compared to the reference 40 to 54 years cohort, though this was significant for only some of the days supply imputation method/patient age combinations. Medication possession levels appeared to peak at 65 to 74 years and then decline for patients 75 years and older. Having an ocular hypertension diagnosis had no significant linear association with medication possession for any of the three days supply estimation methods.

**Table 4:** Regression analyses of persistence and days coverage on index drug

	Method of imputing days supply					
	**Unadjusted**		**2.0-2.1 variable factor^†^**		**2.0 Constant Factor** **45 Minimum^‡^**	
	
Variable*	**Odds** **ratio**	**P value**	**Odds** **ratio**	**P value**	**Odds ratio**	**P value**

**Logistic regression of persistence on index drug**						

Bimatoprost	0.66	< 0.001	0.74	< 0.001	0.71	< 0.001
Travoprost	0.66	< 0.001	0.64	< 0.001	0.66	< 0.001
Female	0.99	0.889	0.97	0.545	0.96	0.423
Age 55 to 64 years	1.12	0.088	1.19	0.003	1.17	0.010
Age 65 to 74 years	1.21	0.009	1.27	< 0.001	1.24	0.001
Age 75+ years	1.10	0.204	1.14	0.057	1.16	0.025
Ocular hypertension	0.99	0.886	1.00	0.920	1.00	0.933

**Linear regression of days coverage on index drug**						

	**Coefficient**	**P value**	**Coefficient**	**P value**	**Coefficient**	**P value**

Bimatoprost	-21.60	< 0.001	-21.03	< 0.001	-28.85	< 0.001
Travoprost	-32.84	< 0.001	-42.11	< 0.001	-39.44	< 0.001
Female	0.33	0.866	-0.56	0.833	-0.42	0.873
Age 55 to 64 years	12.67	< 0.001	17.17	< 0.001	16.21	< 0.001
Age 65 to 74 years	22.40	< 0.001	26.50	< 0.001	26.09	< 0.001
Age 75+ years	10.87	< 0.001	11.18	0.004	14.49	< 0.001
Ocular hypertension	2.31	0.241	-0.01	0.997	-0.17	0.950
Intercept	128.94	< 0.001	226.28	< 0.001	235.24	< 0.001

*Reference case is latanoprost as index drug; male; age 40 to 54 years; diagnostic-coded medical visit for preglaucoma or ocular hypertension during 180-day preindex period.

^†^Imputed days supply is unadjusted days supply multiplied by 2.0 (latanoprost, travoprost) or 2.1 (bimatoprost).

^‡^Imputed days supply is unadjusted days supply multiplied by 2.0 (all prostaglandins), with a minimum imputed estimate of 45 days.

Results from the linear regression analysis for days covered (Table 4) showed similar findings. In this adjusted model, days covered on therapy during the first therapy year were 21 to 29 days lower for bimatoprost and 33 to 42 days lower for travoprost (p ≤ 0.001 for all comparisons) when compared to the reference latanoprost. Bimatoprost had 11 to 21 greater days covered on therapy than travoprost (p < 0.001 to 0.013). Higher days covered for the over-55 cohorts compared to the 40- to 54-year-old cohort was significant for all comparisons (p < 0.001 for all). Again, days covered levels appeared to peak at 65 to 74 years and then decline for patients 75 years and older. As with medication possession, ocular hypertension had no significant linear association with days covered for any of the three days supply estimation methods.

## Discussion

A failure of even a minority of patients to achieve optimal persistence for the progressive and often asymptomatic disease of glaucoma can have important health consequences. Our study of treatment-naive patients dispensed bimatoprost, latanoprost, or travoprost showed that 1-year persistence is less than optimal for many patients who are new to prostaglandin therapy. Even the most liberal methods for estimating days supply from the prescription claim (2.0-2.1 Variable Factor and 2.0 Constant Factor/45 Minimum) showed that only 47 to 48% of patients had medication possession at 1 year from the start of therapy across treatments. Similarly, when days covered was expressed as a ratio with the 358-day follow-up period, these more liberal estimates of days supply showed that patients had days covered with prostaglandins for only 64% (228/358) to 66% (236/358) of the days in the first follow-up year. Of these two persistence measures, days covered on therapy may be a more reliable measure of continued therapy usage than medication possession at a fixed point in time since the former includes all prescription claims in the first therapy year while the latter assesses only the expected therapy coverage delivered by the last prescription fill within the first therapy year.

A recent analysis by Lee and associates [24] evaluated persistence across all prostaglandin agents using a gap analysis methodology in which periods of 45, 60, and 120 days were permitted between fills before a gap in therapy was identified. Using these assumptions, 11%, 29%, and 78% of patients, respectively, had no gaps in therapy, and 33%, 53%, and 87%, respectively, had 30 days or fewer off therapy annually. In contrast to our suboptimal findings for prostaglandin persistence, this analysis found that only a minority of patients receiving prostaglandins generally experienced gaps in therapy over the course of a year. However, in that study, patients were not required to be new to prostaglandin therapy and were required, for inclusion, to have received a refill of the same prostaglandin within 1 year after the index fill. As found in the current analysis, exclusion of subjects from analysis who failed to refill their initial prostaglandin would remove a substantial number of potential subjects (45 to 63% of the total sample in our study) and cause persistence rates to appear much higher than otherwise. Lack of persistence among a large and important group of subjects is thus ignored; such a protocol would "essentially exclud [e] patients who discontinued therapy for reasons such as lack of efficacy or side effects" [28].

### Early failure as a screening tool for lack of persistence

Early failures could be related to any of a variety factors, e.g., adverse effects, switching of agents, use of medication samples, cost. Since we found that excluding "early failures" strongly improved persistence rates for the retained sample, we conducted an unplanned follow-up analysis to evaluate whether a simple "early failure" test could be used as a practical tool to screen patients who were likely to be nonpersistent. First, we coded each patient as being an early failure (ie, failed to refill within 90 days) or not and then determined whether he or she had medication possession at 358 days and, separately, whether he or she achieved at least 270 days covered (ie, 75% coverage throughout the year).

Results indicated modest sensitivity (53 to 70%) and specificity (63 to 70%) of this "early failure" rule for predicting medication possession and days covered when patients receiving any quantity of therapy on the index date were included (Table 5). However, when initial fills on the index date were limited to single bottles of the smallest quantity available, 2.5 mL, which was dispensed for 68% of patients studied, sensitivity declined somewhat but specificity improved considerably, especially when estimating whether patients had achieved ≥ 270 days covered (specificity ranged from 90 to 99%). Thus, evidence of the utility of this test was greatest for *ruling in *or identifying those who are especially unlikely to achieve at least 75% coverage of days supply throughout the year for those patients who started therapy with single fills of 2.5 mL bottles and did not refill within 90 days. The *rule-out *converse, predicting whether a patient who actually does refill within 90 days is likely to successfully complete at least 75% days coverage over the year, is much less certain from this test. When compared among prostaglandins, these "early failure" rates for single fills of 2.5 mL bottles were 47% for bimatoprost, 37% for latanoprost, and 49% for travoprost (p < 0.001, chi-square). Thus, these findings roughly parallel the pattern of inter-prostaglandin comparisons for persistence shown in Table 3.

**Table 5:** Sensitivity/specificity of 90 days/persistence

	Any quantity fills		Single bottle gills of 2.5 mL	
Method of imputing days supply	Sensitivity	Specificity	Sensitivity	Specificity
**Medication possession**				
Unadjusted days supply	56%	63%	47%	79%
2.0-2.1 Variable Factor*	60%	60%	53%	77%
2.0 Constant Factor/45				
Minimum^†^	61%	60%	54%	77%

**Days covered**				
Unadjusted days supply	53%	68%	43%	99%
2.0-2.1 Variable Factor*	67%	69%	61%	90%
2.0 Constant Factor/45				
Minimum^†^	70%	70%	65%	90%

*Imputed days supply is unadjusted days supply multiplied by 2.0 (latanoprost, travoprost) or 2.1 (bimatoprost).

^†^Imputed days supply is unadjusted days supply multiplied by 2.0 (all prostaglandins), with a minimum imputed estimate of 45 days.

In this study, we relied on claims data to identify refills. Ophthalmologists are unlikely to have access to such data but would need to rely on patient self-reports regarding refill history. Patient self-reports regarding compliance or persistence should be interpreted cautiously [33]. Still, if a patient query tool could be developed that was largely accurate for revealing refill history early in therapy, it might serve well as a screening tool to identify patients who are unlikely to achieve appropriate therapeutic coverage on their index prostaglandin agent and require encouragement towards persistence or a physician-directed change in therapy. Further, such a tool could be used to reinforce persistence through repeated administration by the ophthalmologist over the cycle of follow-up visits needed to establish intraocular pressure control.

### Comparisons among prostaglandin analogues

Regression models strongly support a persistence advantage for latanoprost compared to bimatoprost and travoprost, even after adjusting for differences in covariates. Similar findings favouring for latanoprost were found in an earlier study [20] that used a survival analysis methodology: patients treated with bimatoprost and travoprost were 38 and 36% more likely to discontinue therapy, respectively, when compared to latanoprost users (p < 0.001 for each comparison). In this earlier work [20], authors suggested differences in rates of hyperemia among these three agents as a potential explanation for differences in persistence. In contrast, Wilensky and associates [27] found that when "early failures" were excluded from analysis, latanoprost users had 10 fewer days of adherent therapy compared to bimatoprost (p < 0.05) with no significant difference between bimatoprost and travoprost users.

We expected that patients with a medical visit coded for ocular hypertension/preglaucoma would have lower medication possession and days covered than the remaining patients. Nordstrom and associates [23] found evidence of a modest effect (odds ratio = 0.92, p < 0.05) in this direction in their comparison of persistence by therapeutic class of ocular hypotensives used. We presumed that glaucoma suspects would be more likely to discontinue therapy than patients with a glaucoma diagnosis. However, neither regression model showed evidence of such a relationship. With regard to age, results suggest that persistence improves with age but that improvement peaks at ages 65 to 74 and declines thereafter.

### Further Research Needed

Although in the current study we have focused on a specific dimension of adherence, namely estimation techniques, further research is needed regarding the scope of persistence problems in glaucoma, comparisons of persistence rates among agents and patient groups, the link between persistence and patient outcomes, and evaluation of effective methods to improve persistence among patients being treated for glaucoma. For instance, we have learned from more extensive studies of other therapeutic classes, such as statins, that persistence is generally poor [34], that differences in persistence likely exist among agents used to treat target conditions [34,35], that high adherence is related to worsened medical outcomes, such as development of congestive heart failure [36], and that health provider interventions can be shown to improve adherence to statins [37]. In addition to exploring alternate methods for assessing adherence, such as electronic monitoring, further research should explore methods of improving persistence and adherence. To date, little empirical research has evaluated the impact of educational or reminder interventions to improve persistence and adherence of glaucoma therapy.

### Limitations and strengths

This study has a number of limitations. First, as is common to all claims-based assessments, incomplete claims data (eg, out-of-pocket purchases, use of medication samples) could have influenced findings. Second, there is no "gold standard" for estimating days supply from prescription claim records, particularly if multiple sizes or more than one bottle of medication are dispensed in a fill, so we relied on an assessment of days supply through a sensitivity analysis approach. Third, as Fiscella and associates [32] and Mick and associates [31] have shown, the number of drops available in 2.5 mL bottles of bimatoprost exceeds those available in the latanoprost and travoprost bottles due to overfill. The 2.1 (bimatoprost) vs 2.0 (latanoprost and travoprost) adjustment factor used in the days supply imputation "2.0-2.1 Variable Factor" may not have adequately captured the effect of this difference. Further, it is possible that due to differences in overfill some patients using certain prostaglandin products might have either delayed refills due to the "extra" amount of drug available at months' end due to overfill, or might have exhausted their drug supply before months' end and chosen not to refill until the following month, when they would have become eligible for refills under terms of their pharmacy insurance coverage. Fourth, several adjustments for differences among prostaglandin therapy cohorts were made in regression models; factors unavailable in a claims database, such as baseline intraocular pressure or human difficulty in applying a single daily drop to each eye, may have influenced findings but are unavailable in a claims database. Fifth, "days covered" as an adherence measure differs from "coverage" in a pharmacologic sense, since the latter more fully considers the dose-response relationships that may vary among patients and across pharmacotherapies. Finally, possession of the drug or an adequate days supply derived from initial fills and refill data does not directly address compliance behavior (following administration instructions) since a patient may not always properly use the medication while it is in his/her possession. A patient may appear to be persistent or adherent using the methods we applied, yet inconsistent dosing schedules or administration of drops that miss the eye, as has been documented in some studies [38,39], may mean that some patients are not consistently receiving reliable coverage from the prescribed prostaglandins to effectively achieve consistent intraocular pressure control. Use of an electronic monitoring method may provide a more accurate alternative to pharmacy dispensing data and can yield further insight into compliance behavior. When using electronic monitoring for assessing adherence of antihypertensive therapy in Phase IV clinical trials, Vrijens et al. [40] showed that 10% of all scheduled doses were omitted by subjects: 42% of these were of a single day's dose, whereas 43% were part of a sequence of several days (i.e. "drug "holidays"). In glaucoma research, Robin et al. [41] used electronic monitoring of topical ocular hypotensives to capture a breadth of measures not possible with simple observations of prescription fills, including dosing errors, coverage as measured by daily adherence to scheduled times of administration, intervals or gaps between doses, and percent of doses actually taken.

This study also had a number of strengths. The naturalistic setting described by the claims data and the large number of patients supported the generalisability of findings and provided statistical power. The methods used to assess persistence are based on concepts (medication possession and days covered) that have been evaluated for many other medical conditions.

## Conclusions

Our findings strongly suggest that persistence with ocular prostaglandin therapy remains a problem for many patients and should be a focus of educational efforts by ophthalmologists. This problem was not apparent in some earlier persistence studies since excluding patients from analyses based on postindex experience would be expected to overestimate persistence rates. In general, latanoprost users had greater odds of achieving medication possession and more days covered during the first therapy year than those prescribed bimatoprost or travoprost, suggesting that choice of therapy may be an important consideration to improve persistence.

## Competing interests

Drs. Reardon and Schwartz are consultants to Pfizer Inc. Mr. Kotak is an employee of Pfizer Inc.

## Authors' contributions

All authors made substantial contributions to the conception and design of the study. GR and SK acquired, analyzed, and interpreted the data. All authors were involved in drafting the manuscript and/or revising it critically. All authors read and approved the final manuscript.

## Pre-publication history

The pre-publication history for this paper can be accessed here:

http://www.biomedcentral.com/1471-2415/10/5/prepub
